# Neural Speech Tracking Contribution of Lip Movements Predicts Behavioral Deterioration When the Speaker's Mouth Is Occluded

**DOI:** 10.1523/ENEURO.0368-24.2024

**Published:** 2025-02-03

**Authors:** Patrick Reisinger, Marlies Gillis, Nina Suess, Jonas Vanthornhout, Chandra Leon Haider, Thomas Hartmann, Anne Hauswald, Konrad Schwarz, Tom Francart, Nathan Weisz

**Affiliations:** ^1^Department of Psychology, Centre for Cognitive Neuroscience, Paris-Lodron-University of Salzburg, Salzburg 5020, Austria; ^2^Experimental Oto-Rhino-Laryngology, Department of Neurosciences, Leuven Brain Institute, KU Leuven, Leuven 3000, Belgium; ^3^MED-EL GmbH, Innsbruck 6020, Austria; ^4^Neuroscience Institute, Christian Doppler University Hospital, Paracelsus Medical University Salzburg, Salzburg 5020, Austria

**Keywords:** audiovisual speech, lip movements, MEG, neural tracking, temporal response functions, TRF

## Abstract

Observing lip movements of a speaker facilitates speech understanding, especially in challenging listening situations. Converging evidence from neuroscientific studies shows stronger neural responses to audiovisual stimuli compared with audio-only stimuli. However, the interindividual variability of this contribution of lip movement information and its consequences on behavior are unknown. We analyzed source-localized magnetoencephalographic responses from 29 normal-hearing participants (12 females) listening to audiovisual speech, both with and without the speaker wearing a surgical face mask, and in the presence or absence of a distractor speaker. Using temporal response functions to quantify neural speech tracking, we show that neural responses to lip movements are, in general, enhanced when speech is challenging. After controlling for speech acoustics, we show that lip movements contribute to enhanced neural speech tracking, particularly when a distractor speaker is present. However, the extent of this visual contribution to neural speech tracking varied greatly among participants. Probing the behavioral relevance, we demonstrate that individuals who show a higher contribution of lip movements in terms of neural speech tracking show a stronger drop in comprehension and an increase in perceived difficulty when the mouth is occluded by a surgical face mask. In contrast, no effect was found when the mouth was not occluded. We provide novel insights on how the contribution of lip movements in terms of neural speech tracking varies among individuals and its behavioral relevance, revealing negative consequences when visual speech is absent. Our results also offer potential implications for objective assessments of audiovisual speech perception.

## Significance Statement

In complex auditory environments, simultaneous conversations pose a challenge to speech comprehension. We investigated on a neural level how lip movements aid in such situations and what the behavioral consequences are, especially when lip information is occluded with a face mask. Using magnetoencephalographic responses from participants listening to audiovisual speech, we show that observing lip movements enhances neural speech tracking and participants who rely more on lip movements show behavioral deterioration when the speaker wears a face mask. Remarkably, this is not the case when no face mask was worn by the speaker. Our findings reveal interindividual differences in the contribution of lip movements to neural speech tracking, with potential applications in objective assessments of audiovisual speech perception.

## Introduction

Face masks are an important tool in preventing the spread of contagious diseases such as COVID-19 (Chu et al., 2020; Suñer et al., 2022). However, as many have subjectively experienced firsthand, the use of face masks also impairs speech perception, and not only by attenuating sound. More importantly, they occlude facial expressions, such as lip movements (Brown et al., 2021; Rahne et al., 2021), that provide visual information for a relevant speech stream. This is particularly critical when speech is challenging, such as in the classic cocktail party situation, where conversations are happening simultaneously (Cherry, 1953). Ideally, visual information is available to aid in such situations, with numerous studies demonstrating that visual speech features enhance the understanding of degraded auditory input (Sumby and Pollack, 1954; Grant and Seitz, 2000; Ross et al., 2007; Remez, 2012). Among visual speech features, lip movements are the most important, playing a crucial role in the perception of challenging speech (Erber, 1975; Peelle and Sommers, 2015). However, substantial interindividual differences in lip-reading performance among normal, as well as hearing-impaired, populations have been shown in previous studies (Suess et al., 2022b; for a review see Summerfield et al., 1992). Despite our imperfect lip-reading abilities, the human brain effectively uses lip movements to facilitate the perception of challenging speech, with the neural mechanisms and regions involved still under debate (Ross et al., 2022; Zhang and Du, 2022).

A suitable method for studying the neural responses to audiovisual speech is neural speech tracking (Obleser and Kayser, 2019; Brodbeck and Simon, 2020). This method typically aims to predict the neural response to one or more stimulus features via so-called temporal response functions (TRFs; Crosse et al., 2021). The TRF approach has so far extended our understanding of speech processing from acoustic features (Lalor et al., 2009) to higher-level linguistic features (Broderick et al., 2018; Brodbeck et al., 2018a; Gillis et al., 2021).

Previous studies have shown beneficial effects of visual speech on the representation of speech in the brain. A magnetoencephalography (MEG) study by Park et al. (2016) showed enhanced entrainment between lip movements and speech-related brain areas when congruent audiovisual speech was presented. Other studies have shown that the incorporation of visual speech enhances the ability of the brain to track acoustic speech (Golumbic et al., 2013; Crosse et al., 2015, 2016b). Interestingly, when silent lip movements are presented, the visual cortex also follows the unheard acoustic speech envelope (Hauswald et al., 2018) or unheard spectral fine details (Suess et al., 2022a). Despite these findings, two questions remain unanswered: First, it is unknown to which extent individuals vary in their unique contribution of lip movements to neural speech tracking. Given the aforementioned interindividual differences in lip-reading performance, we hypothesize a high degree of variability in this contribution. Second, it is unknown if the unique contribution of lip movements is of behavioral relevance, as, for example, when the lips are occluded with a face mask, as has been common during the COVID-19 pandemic. Given the negative impact of face masks on behavioral measures (Rahne et al., 2021; Toscano and Toscano, 2021; Truong et al., 2021), we expect the following relationship: Individuals who show a higher unique contribution of lip movements should also show poorer behavioral outcomes when no lip movements are available, as they are deprived of critical visual information.

**Figure 1.**
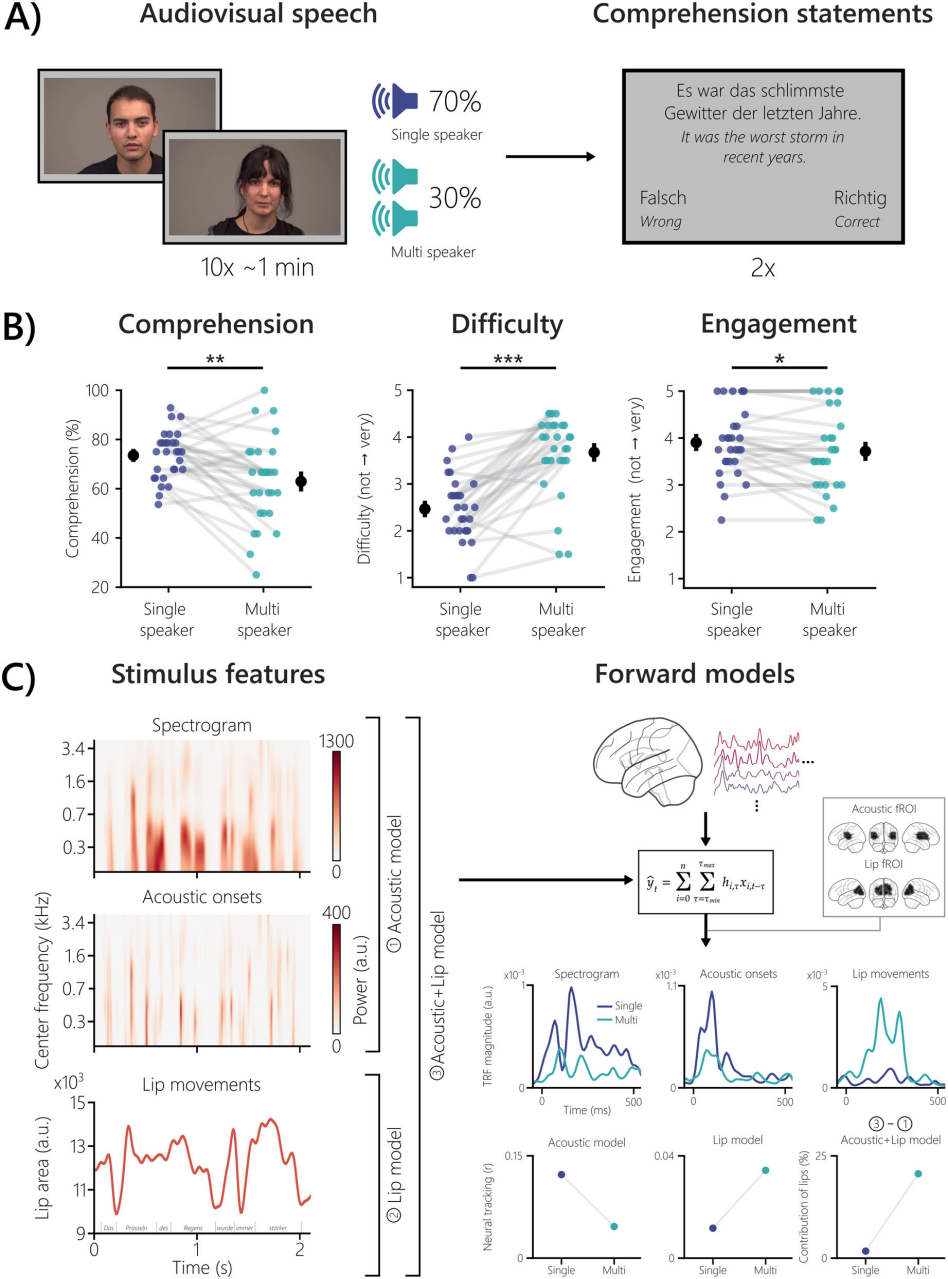
Experimental design, behavioral results, and analysis framework. ***A***, Each block consisted of 10 ∼1 min trials of continuous audiovisual speech by either a female or male speaker (single-speaker condition). In 30% of these 10 trials, a same-sex audio–only distractor speaker was added (multispeaker condition). After every block, two comprehension statements had to be rated as correct or wrong. ***B***, Comprehension was lower in the multispeaker condition than in the single-speaker condition (*p* = 0.003; *r*_C_ = 0.64). Subjective difficulty ratings, reported on a five-point Likert scale, were higher in the multispeaker condition (*p* = 9.00 × 10^−6^; *r*_C_ = −0.95). The reported engagement was lower in the multispeaker condition (*p* = 0.024; *r*_C_ = 0.62). The black dots represent the mean, and the bars represent the standard error of the mean (SEM). ***C***, Three stimulus features (spectrogram, acoustic onsets, and lip movements) extracted from the audiovisual stimuli are shown for an example sentence. Higher values in the lip area unit represent a wider opening of the mouth and vice versa. Three forward models were calculated: (1) one using only acoustic features, (2) one using only lip movements, and (3) one combining all features. Together with the corresponding source-localized MEG data, the boosting algorithm was used to calculate the models. Exemplary minimum-norm source estimates are shown for a representative participant. The resulting TRFs (a.u.) and neural tracking (expressed as Pearson's *r*) were analyzed in fROIs, obtained either via the acoustic or lip model of the multispeaker condition. The TRFs and prediction accuracies shown are from a representative participant reflecting the group-level results. To obtain the unique contribution of lip movements, we controlled acoustic features by subtracting the prediction accuracies in an acoustic + lip fROI of the acoustic model from the combined model. The unique contribution of lip movements was expressed as a percentage change. *p < 0.05; **p < 0.01; ***p < 0.001.

Using MEG and an audiovisual speech paradigm with one or two speakers, we probed the unique contribution of lip movements and its behavioral relevance. Utilizing a state-of-the-art neural tracking framework with source-localized TRFs (Fig. 1*C*), we show that lip movements elicit stronger neural responses when speech is difficult compared with when it is clear. Additionally, we show that the neural tracking of lip movements is enhanced in multispeaker settings. When controlled for acoustic speech features, the obtained unique contribution of lip movements is, in general, more enhanced in the multispeaker condition, with substantial interindividual variability. Using Bayesian modeling, we show that acoustic speech tracking is related to behavioral measures. Crucially, we demonstrate that individuals who show a higher unique contribution of lip movements show a stronger drop in comprehension and report a higher subjective difficulty when the mouth is occluded by a surgical face mask. In terms of neural tracking, our results suggest that individuals show a unique contribution of lip movements in a highly variable manner. We also establish a novel link between the neural unique contribution of visual speech and behavior when no lip movement information is available.

## Materials and Methods

### Participants

The data were collected as part of a recent study (Haider et al., 2022), in which 30 native speakers of German participated. One participant was excluded because signal source separation could not be applied to the MEG dataset due to file corruption. This led to a final sample size of 29 participants aged between 22 and 41 years (12 females; *M*_age_ = 26.79 years; SD_age_ = 4.87 years). All participants reported normal vision and hearing (thresholds did not exceed 25 dB HL at any frequency from 125 to 8,000 Hz), the latter verified by a standard clinical audiometer (AS608 Basic; Interacoustics A/S). Additional exclusion criteria included nonremovable magnetic objects and any psychiatric or neurologic history. All participants signed an informed consent and were reimbursed at a rate of 10 € per hour. The experimental protocol was approved by the ethics committee of the Paris-Lodron-University of Salzburg and was conducted in accordance with the Declaration of Helsinki.

### Stimuli and experimental design

The experimental procedure was implemented in MATLAB 9.10 (The MathWorks) using custom scripts. Presentation of stimuli and response collection was achieved with the Objective Psychophysics Toolbox (o_ptb; Hartmann and Weisz, 2020), which adds a class-based abstraction layer onto the Psychophysics Toolbox version 3.0.16 (Brainard, 1997; Pelli, 1997; Kleiner et al., 2007). Stimuli and triggers were generated and emitted via the VPixx system (DATAPixx2 display driver, PROPixx DLP LED projector, RESPONSEPixx response box; VPixx Technologies). Videos were back-projected onto a translucent screen with a screen diagonal of 74 cm (∼110 cm in front of the participants), with a refresh rate of 120 Hz and a resolution of 1,920 × 1,080 pixels. Timings were measured with the Black Box ToolKit v2 (The Black Box ToolKit) to ensure accurate stimulus presentation and triggering.

The audiovisual stimuli were excerpts from four German stories, two of each read out loud by a female or male speaker (female: “Die Schokoladenvilla - Zeit des Schicksals. Die Vorgeschichte zu Band 3” by Maria Nikolai , “Die Federn des Windes” by Manuel Timm; male: “Das Gestüt am See. Charlottes großer Traum” by Paula Mattis and “Gegen den Willen der Väter” by Klaus Tiberius Schmidt). A Sony NEX-FS100 (Sony) camera with a sampling rate of 25 Hz and a RØDE NTG2 microphone (RØDE Microphones) with a sampling rate of 48 kHz were used to record the stimuli. Each of the four stories was recorded twice, once with and once without a surgical face mask (type IIR three-layer disposable medical mask). These eight videos were cut into 10 segments of ∼1 min each (*M* = 64.29 s; SD = 4.87 s), resulting in 80 videos. In order to rule out sex-specific effects, 40 videos (20 with a female speaker and 20 with a male speaker) were presented to each participant. The speakers’ syllable rates were analyzed using Praat (Boersma, 2001; de Jong and Wempe, 2009) and varied between 3.7 and 4.6 Hz (*M* = 4.1 Hz). The audio-only distractor speech consisted of prerecorded audiobooks (Schubert et al., 2023), read by either a female or a male speaker. All audio files were normalized using ffmpeg-normalize version 1.19.1 (running on Python 3.9.7) with default options.

Before the experiment, a standard clinical audiometry was performed (for details, see above, Participants). The MEG measurement started with a 5 min resting-state recording (not analyzed in this manuscript). Next, the participant's individual hearing threshold was determined in order to adjust the stimulation volume. If the participant reported that the stimulation was not loud enough or comfortable, the volume was manually adjusted to the participant's requirements. Hearing threshold levels ranged from −91.76 db (RMS) to −68.78 db (RMS) [*M* = −80.57 db (RMS); SD = 4.20 db (RMS)].

The actual experiment consisted of four stimulation blocks, one for each of the four stories, with two featuring each sex. Each story was presented as a block of 10 ∼1 min trials (ranging from 0.93 to 1.27 min) in a chronological order to preserve the story content (Fig. 1*A*). In every block, a same-sex audio–only distractor speaker was added to three randomly selected trials, with a 5 s delay and volume equal to the target speaker. The resulting ratio of 30% multispeaker trials and 70% single-speaker trials per block was chosen because of a different data analysis method in Haider et al. (2022). The distractor speech started with a delay of 5 s to give participants time to attend the target speaker. In two randomly selected blocks, the target speaker wore a face mask (only the corresponding behavioral data were used here; see below, Statistical analysis and Bayesian modeling). Two unstandardized correct or wrong statements about semantic content were presented after each trial to assess comprehension and to maintain attention (Fig. 1*A*). On four occasions in each block, participants also rated subjective difficulty and engagement on a five-point Likert scale (not depicted in Fig. 1*A*). The participants responded by pressing buttons. The blocks were presented randomly, and the total duration of the experiment was ∼2 h, including preparation.

### MEG data acquisition and preprocessing

Before entering the magnetically shielded room, five head position indicator (HPI) coils were applied on the scalp. Electrodes for electrooculography (vertical and horizontal eye movements) and electrocardiography were also applied (recorded data not used here). Fiducial landmarks (nasion and left/right preauricular points), the HPI locations, and ∼300 head shape points were sampled with a Polhemus FASTRAK digitizer (Polhemus).

Magnetic brain activity was recorded with a Neuromag Triux whole-head MEG system (MEGIN Oy, Espoo, Finland) using a sampling rate of 1,000 Hz (hardware filters, 0.1–330 Hz). The signals were acquired from 102 magnetometers and 204 orthogonally placed planar gradiometers at 102 different positions. The system is placed in a standard passive magnetically shielded room (AK3b; Vacuumschmelze).

A signal space separation (Taulu and Kajola, 2005; Taulu and Simola, 2006) algorithm implemented in MaxFilter version 2.2.15 provided by the MEG manufacturer was used. The algorithm removes external noise from the MEG signal (mainly 16.6, and 50 Hz, plus harmonics) and realigns the data to a common standard head position (to [0 0 40] mm, -*trans default* MaxFilter parameter) across different blocks, based on the measured head position at the beginning of each block.

Preprocessing of the raw data was done in MATLAB 9.8 using the FieldTrip toolbox (revision f7adf3ab0; Oostenveld et al., 2011). A low-pass filter of 10 Hz (hamming-windowed sinc FIR filter; onepass-zerophase; order, 1320; transition width, 2.5 Hz) was applied, and the data were downsampled to 100 Hz. Afterward, a high-pass filter of 1 Hz (hamming-windowed sinc FIR filter; onepass-zerophase; order, 166; transition width, 2.0 Hz) was applied.

Independent component analysis (ICA) was used to remove eye and cardiac artifacts (data were filtered between 1 and 100 Hz; sampling rate, 1,000 Hz) via the infomax algorithm (“runica” implementation in EEGLAB; Bell and Sejnowski, 1995; Delorme and Makeig, 2004) applied to a random block of the main experiment. Prior to the ICA computation, we performed a principal component analysis with 50 components in order to ease the convergence of the ICA algorithm. After visual identification of artifact-related components, an average of 2.38 components per participant were removed (SD = 0.68).

The cleaned data were epoched into trials that matched the length of the audiovisual stimuli. To account for an auditory stimulus delay introduced by the tubes of the sound system, the data were shifted by 16.5 ms. In the multispeaker condition, the first 5 s of data were removed to match the onset of the distractor speech. The last eight trials were removed to equalize the data length between the single-speaker and multispeaker conditions. To prepare the data for the following steps, the trials in each condition were concatenated. This resulted in a data length of ∼6 min per condition.

### Source localization

Source projection of the data was done with MNE-Python 1.1.0 running on Python 3.9.7 (Gramfort et al., 2013, 2014). A semiautomatic coregistration pipeline was used to coregister the FreeSurfer “fsaverage” template brain (Fischl, 2012) to each participant's head shape. After an initial fit using the three fiducial landmarks, the coregistration was refined with the iterative closest point algorithm (Besl and McKay, 1992). Head shape points that were >5 mm away from the scalp were automatically omitted. The subsequent final fit was visually inspected to confirm its accuracy. This semiautomatic approach performs comparably to manual coregistration pipelines (Houck and Claus, 2020).

A single-layer boundary element model (BEM; Akalin-Acar and Gençer, 2004) was computed to create a BEM solution for the “fsaverage” template brain. Next, a volumetric source space with a grid of 7 mm was defined, containing a total of 5,222 sources (Kulasingham et al., 2020). In order to remove nonrelevant regions and shorten computation times, subcortical structures along the midline were removed, reducing the source space to 3,053 sources (similar to Das et al., 2020). Subsequently, the forward operator (i.e., lead field matrix) was computed using the individual coregistrations, the BEM, and the volume source space.

Afterward, the data were projected to the defined sources using the minimum norm estimate (MNE) method (Hämäläinen and Ilmoniemi, 1994). MNE is known to be biased toward superficial sources, which can be reduced by applying depth weighting with a coefficient between 0.6 and 0.8 (Lin et al., 2006). For creating the MNE inverse operator, depth weighting with a coefficient of 0.8 was used (Brodbeck et al., 2018a). The required noise covariance matrix was estimated with an empty-room MEG recording relative to the participant's measurement date with the same preprocessing settings as the MEG data of the actual experiment (see above, MEG data acquisition and preprocessing). The MNE inverse operator was then applied to the concatenated MEG data with ℓ2 regularization [signal-to-noise ratio (SNR) = 3 dB, $\lambda^{2}\;=\;\frac{1}{{\mathrm{SNR}}^{2}}$] and three free-orientation dipoles orthogonally at each source.

### Extraction of stimulus features

Since the focus of this study is on audiovisual speech, we extracted acoustic (spectrograms and acoustic onsets) and visual (lip movements) speech features from the stimuli (Fig. 1*C*). The spectrograms of the auditory stimuli were obtained using the Gammatone Filterbank Toolkit 1.0 (Heeris, 2013), with frequency cutoffs at 20 and 5,000 Hz, 256 filter channels, and a window time of 0.01 s. This toolkit computes a spectrogram representation on the basis of a set of gammatone filters which are inspired by the human auditory system (Slaney, 1998). The resulting filter outputs with logarithmic center frequencies were averaged into eight frequency bands (frequencies <100 Hz were omitted; Gillis et al., 2021). Each frequency band was scaled with exponent 0.6 (Biesmans et al., 2017) and downsampled to 100 Hz, which is the same sampling frequency as the preprocessed MEG data.

Acoustic onset representations were calculated for each frequency band of the spectrograms using an auditory edge detection model (Fishbach et al., 2001). The resulting spectrograms of the acoustic onsets are valuable predictors of MEG responses to speech stimuli (Daube et al., 2019; Brodbeck et al., 2020). A delay layer with 10 delays from 3 to 5 ms, a saturation scaling factor of 30, and a receptive field based on the derivative of a Gaussian window (SD = 2 ms) were used (Gillis et al., 2021). Each frequency band was downsampled to 100 Hz.

The lip movements of every speaker were extracted from the videos with a MATLAB script adapted from Suess et al. (2022a; originally by Park et al., 2016). Within the lip, contour, the area, and the horizontal and vertical axis were calculated. Only the area was used for the analysis, which leads to results comparable with using the vertical axis (Park et al., 2016). The lip area signal was upsampled from 25 to 100 Hz using FFT-based interpolation.

### Forward models

A linear forward modeling approach was used to predict the MEG response to the aforementioned stimulus features (Fig. 1*C*). These approaches are based on the idea that the brain's response to a stimulus is a continuous function in time (Lalor et al., 2006). The boosting algorithm (David et al., 2007), implemented in eelbrain 0.38 (running on Python 3.9.7; Brodbeck et al., 2023), was used to predict MNE source-localized MEG responses to stimulus features (“MNE-boosting”; Brodbeck et al., 2018b). For multiple stimulus features, the linear forward model can be formulated as follows:

$${\hat{y}}_{t}=\overset{n}{\underset{i=0}{\sum }}\;\overset{\tau_{\max}}{\underset{\tau =\tau_{\min}}{\sum }}\;h_{i,\tau }x_{i,t-\tau }.$$

For every n stimulus feature, the algorithm finds an optimal filter kernel h, which is also known as a TRF. When n stimulus feature is >1, h is referred to as multivariate TRF (mTRF). The term τ denotes the delays between the predicted brain response ${\hat{y}}_{t}$ and stimulus feature x (for further details see Brodbeck et al., 2023). TRFs reflect responses to continuous data instead of averaged responses to discrete events (Crosse et al., 2021). For the estimation of the TRFs, the stimulus features and MEG data were normalized (*z*-scored), and an integration window from −100 to 600 ms with a kernel basis of 50 ms Hamming windows was defined. To prevent overfitting, early stopping based on the ℓ2 norm was used. By using fourfold nested cross-validation (two training folds, one validation fold, and one test fold), each partition served as a test set once (Brodbeck et al., 2023). TRFs were estimated for each of the three free-orientation dipoles independently at all 3,053 sources (see above, Source localization). The spectrogram and acoustic onset mTRFs were averaged over the frequency dimension. To account for interindividual anatomical differences, TRFs were spatially smoothed with a Gaussian kernel (SD = 5 mm; Kulasingham et al., 2020). The Euclidean vector norm of the smoothed TRFs was taken, resulting in one TRF per source.

To obtain a measure of neural tracking, we correlated the predicted brain response ${\hat{y}}_{t}$ with the original response to calculate the prediction accuracy and computed as the average dot product over time (expressed as Pearson's correlation coefficient *r*). This correlation indicates that a higher prediction accuracy reflects enhanced neural tracking, meaning that the brain response more closely aligns with the stimulus features (Gillis et al., 2022).

In order to investigate the neural processing of the audiovisual speech features, we calculated three different forward models per condition and participant (see Fig. 1*C* for the analysis framework). The acoustic model consisted of the two acoustic stimulus features (spectrogram and acoustic onsets) and—also applicable to all other models—the corresponding MNE source-localized MEG data. The lip model contained only the lip movements as a stimulus feature. Additionally, a combined acoustic + lip model was calculated to control for acoustic features in a subsequent analysis.

We defined functional regions of interest (fROIs; Nieto-Castanon et al., 2003) by creating labels based on the 90th percentile of the whole-brain prediction accuracies in the multispeaker condition (similar to Suess et al., 2022a). The multispeaker condition was chosen for extracting the fROIs because it potentially incorporates all included stimulus features, due to its higher demand (Golumbic et al., 2013). This was done separately for the acoustic and lip models to map their unique neural sources (Fig. 1*C*). According to the “aparc” FreeSurfer parcellation (Desikan et al., 2006), the acoustic fROI mainly involved sources in the temporal, lateral parietal, and posterior frontal lobes. The superior parietal and lateral occipital lobes made up the majority of the lip fROI. To obtain an audiovisual fROI for the acoustic + lip model, we combined the labels of the acoustic and lip fROIs.

**Figure 2.**
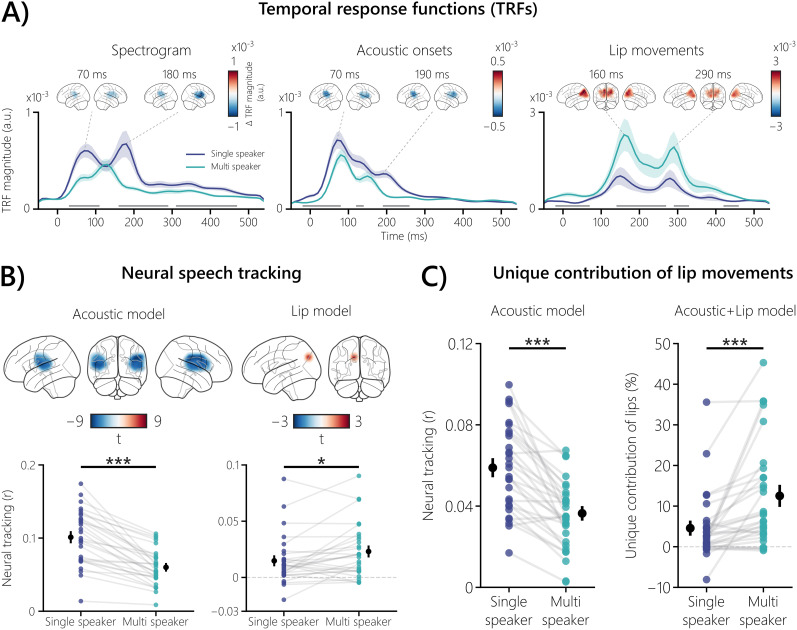
Neural responses to audiovisual speech features, neural speech tracking, and the unique contribution of lip movements. ***A***, The three plots show grand-averaged TRFs for the stimulus features in their respective fROIs and the peak magnitude contrasts (multispeaker vs single speaker) between the two conditions in the involved sources. For the acoustic features, TRF magnitudes were generally enhanced when speech was clear, with significant differences ranging from *p* = 0.004 to *p* < 0.001 (*d* = −0.81 to −1.43). In contrast, the TRF to lip movements showed an enhanced magnitude in the multispeaker condition (*p* = 0.01 to *p* = 0.0005 and effect sizes from *d* = 0.86–0.91). The shaded areas of the respective conditions represent the SEM. Gray bars indicate the temporal extent of significant differences (*p* < 0.05) between the two conditions. ***B***, Neural speech tracking is shown for the nonaveraged fROIs (top brain plots) and averaged fROIs of the acoustic and lip models. Acoustic neural tracking was higher in the single-speaker condition, with significant left- and right-hemispheric differences (both *p* < 0.001 with *d* from −1.30 to −1.47; averaged, *p* = 8.76 × 10^−9^; *d* = −1.30). Lip movements were tracked higher in the multispeaker condition (*p* = 0.037; *d* = 0.51; averaged, *p* = 0.026; *r*_C_ = 0.48). In the averaged plots, the black dots represent the mean, and the corresponding bars the SEM, of the respective condition. ***C***, In a combined acoustic and lip fROI, the acoustic model showed higher neural tracking in the single-speaker condition (*p* = 7.68 × 10^−8^; *d* = 1.18). The unique contribution of lip movements was obtained by subtracting the acoustic model from the acoustic + lip model and expressed as percentage change. Lip movements especially enhanced neural tracking in the multispeaker condition (*p* = 0.00003; *r*_C_ = 0.89). Participants showed high interindividual variability with a unique contribution of lip movements of up to 45.37% but also only a small contribution or no contribution at all. The black dots represent the mean, and the corresponding bars the SEM, of the respective condition. *p < 0.05; **p < 0.01; ***p < 0.001.

For every model, the TRFs in their respective fROI were averaged and, exclusively for Figure 2*A*, smoothed over time with a 50 ms Hamming window. Grand-average TRF magnitude peaks were detected with scipy version 1.8.0 (running on Python 3.9.7; Virtanen et al., 2020) and visualized as a difference between the multi- and single-speaker conditions. To suppress regression artifacts that typically occur (Crosse et al., 2016a), we visualized TRFs between −50 and 550 ms. Prediction accuracies in the fROIs were Fisher *z*-transformed and then averaged, and then the *z* values were back-transformed to Pearson's correlation coefficients (Corey et al., 1998). For the lower panels of each model in Figure 2*B*, the prediction accuracies of the acoustic and lip models were averaged in their respective fROIs. Figures were created with the built-in plotting functions of eelbrain and seaborn version 0.12.0 (running on Python 3.9.7; Waskom, 2021).

In order to answer the question whether or not lip movements enhance neural tracking, a control for acoustic features (spectrograms and acoustic onsets) is needed. This is particularly important due to the intercorrelation of audiovisual speech features (Chandrasekaran et al., 2009; Daube et al., 2019). To investigate the unique contribution of lip movements, we used the averaged prediction accuracies in the audiovisual fROI and subtracted the acoustic model from the acoustic + lip model (for a general overview on control approaches, see Gillis et al., 2022). The resulting unique contribution of lip movements was expressed as percentage change (Fig. 2*C*).

### Statistical analysis and Bayesian modeling

All frequentist statistical tests were conducted with built-in functions from eelbrain and the statistical package pingouin version 0.5.2 (running on Python 3.9.7; Vallat, 2018). The three behavioral measures (comprehension, difficulty, and engagement; Fig. 1*B*) were statistically compared between the two conditions (single speaker and multispeaker) using a Wilcoxon signed-rank test and the matched-pairs rank–biserial correlation *r*_C_ was reported as the effect size (King et al., 2018).

The TRFs corresponding to the three stimulus features (spectrogram, acoustic onsets, and lip movements; Fig. 2*A*) were tested for statistical difference between the two conditions using a cluster-based permutation test with threshold-free cluster enhancement (TFCE; dependent-sample *t* test; 10,000 randomizations; Maris and Oostenveld, 2007; Smith and Nichols, 2009). Due to the previously mentioned TRF regression artifacts, the time window for the test was limited to −50 to 550 ms. Depending on the direction of the cluster, the maximum or minimum *t* value was reported and Cohen's *d* of the averaged temporal extent of the cluster was calculated.

We tested the nonaveraged prediction accuracies in the acoustic and lip fROIs (Fig. 2*B*) with a cluster-based permutation test with TFCE (dependent-sample *t* test, 10,000 randomizations). According to the cluster's direction, the maximum or minimum *t* value was reported, and Cohen's *d* of the cluster's averaged spatial extent was calculated. Additionally, averaged prediction accuracies in the acoustic and lip fROIs were statistically tested with a dependent-sample *t* test, and Cohen's *d* was reported as the effect size. In the audiovisual fROI, the prediction accuracies and unique contribution of lip movements (Fig. 2*C*) were tested with a dependent-sample *t* test, and Cohen's *d* was reported as the effect size. If the data were not normally distributed according to a Shapiro–Wilk test, the Wilcoxon signed-rank test was used, and the matched-pair rank–biserial correlation *r*_C_ was reported as the effect size. The distribution of the contribution of lip movements was assessed using the bimodality coefficient (Freeman and Dale, 2013).

**Figure 3.**
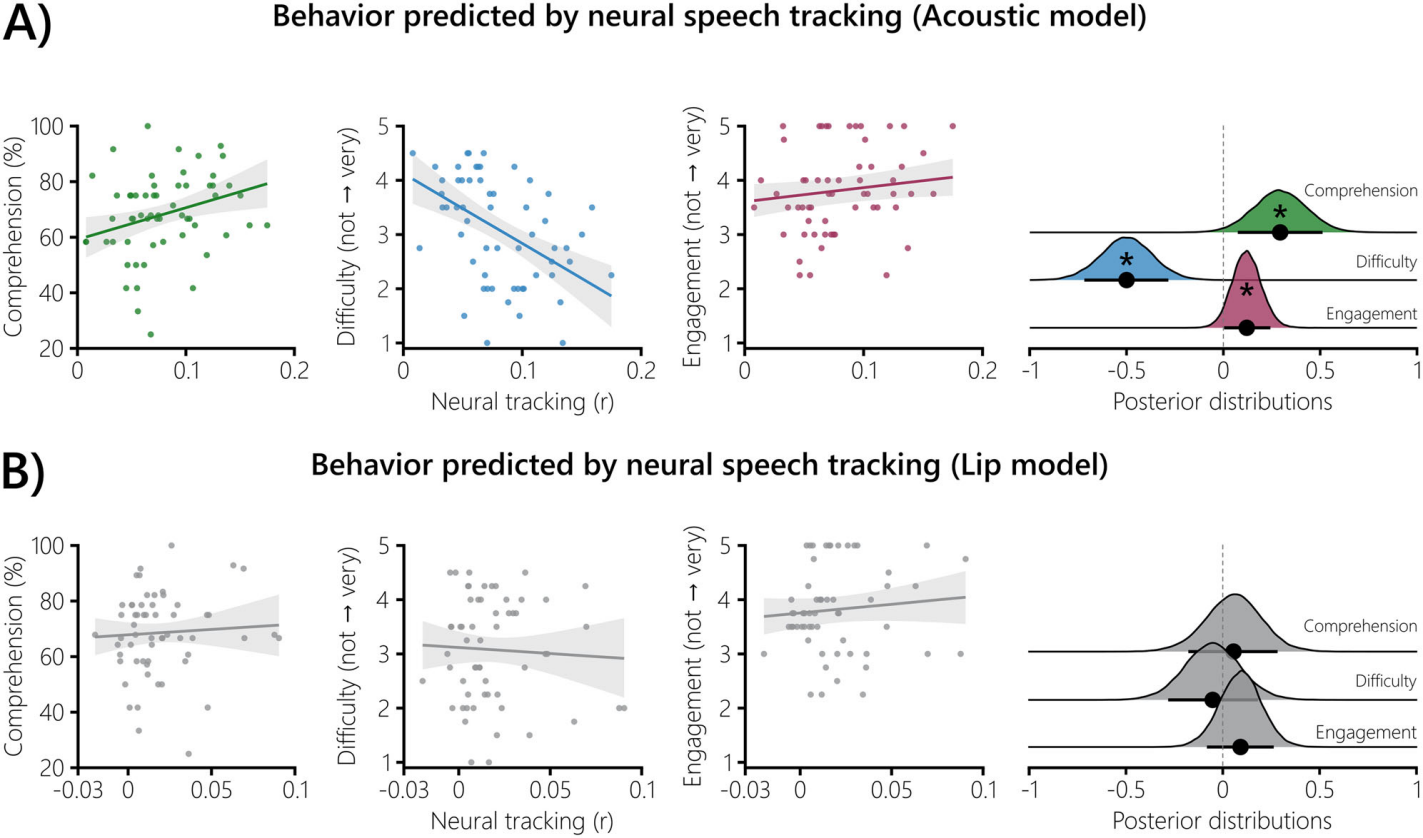
Relating behavior to neural speech tracking. Bayesian multilevel models were fitted to predict the behavioral measures with neural speech tracking. ***A***, Higher acoustic neural speech tracking was linked to higher comprehension, lower difficulty ratings and higher engagement ratings. ***B***, No evidence for an effect was observed for the neural tracking of lip movements. Both panels, The shaded areas show the 89% CIs of the respective model. The distributions on the right show the posterior draws of the three models. The black dots represent the mean standardized regression coefficient *b* of the corresponding model. The corresponding bars show the 89% CI. If zero was not part of the 89% CI, the effect was considered significant (*).

To investigate if neural tracking is predictive for behavior, we calculated Bayesian multilevel models in R version 4.2.2 (R Core Team, 2022) with the Stan-based package brms version 2.18.4 (Bürkner, 2017; Carpenter et al., 2017). Neural tracking (i.e., the averaged prediction accuracies within the respective fROI) was used to separately predict the three behavioral measures (averaged over the same number of trials for all participants). A random intercept was added for each participant to account for repeated measures (single speaker and multispeaker). The models were fitted independently for the acoustic and lip models (Fig. 3). According to the Wilkinson notation (Wilkinson and Rogers, 1973), the general formula was as follows:

behavioralmeasure∼1+neuraltracking+(1|participant).

**Figure 4.**
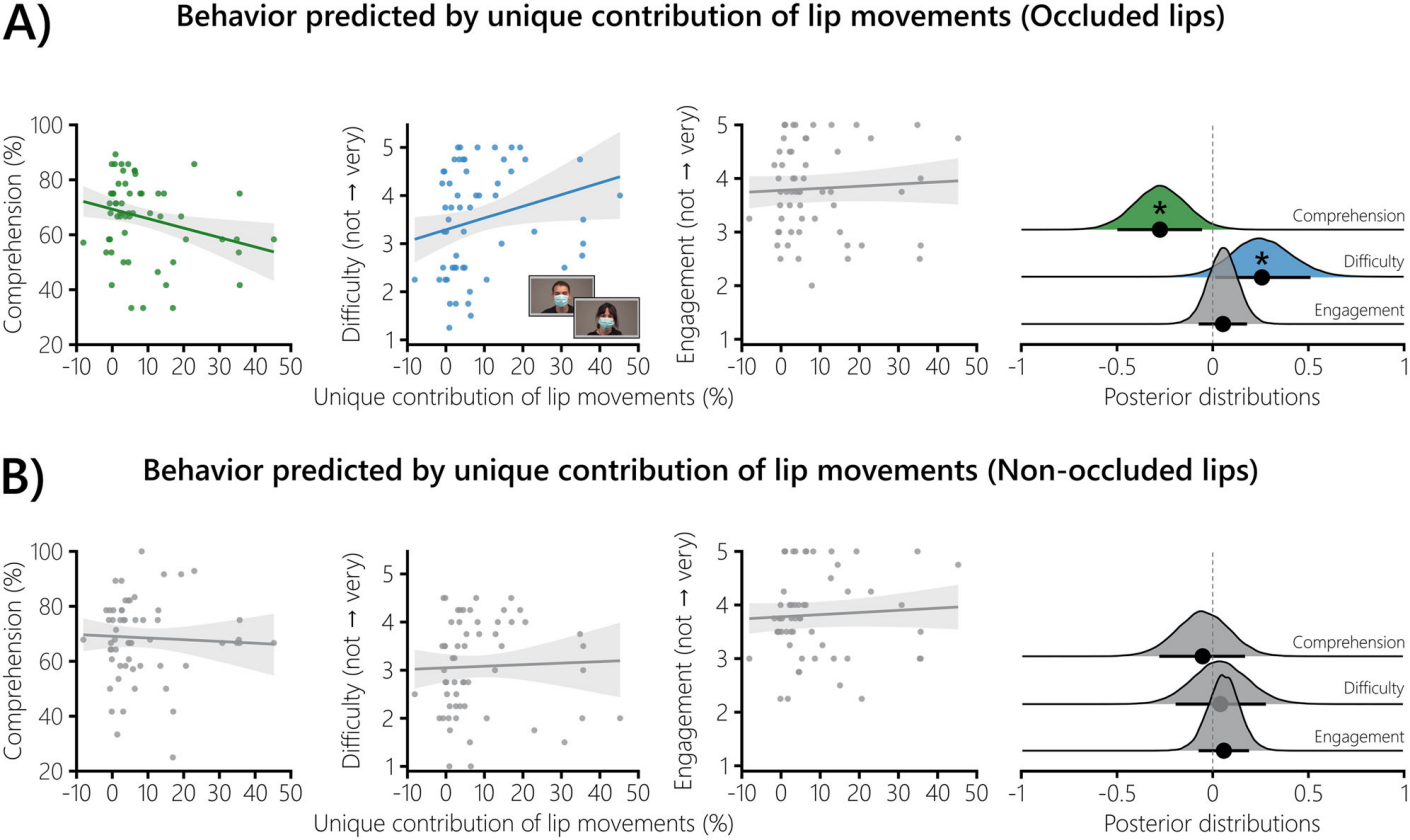
Relating the unique contribution of lip movements to behavior. The unique contribution of lip movements was used to predict the behavioral measures when the lips are occluded or not. ***A***, When the unique contribution of lip movements was high, comprehension was lower, and difficulty was reported higher. No evidence for an effect was observed for the engagement rating. The values of the fitted Bayesian multilevel models are shown with a depiction of the conditions in which the speakers wore a surgical face mask. ***B***, The behavioral measures when the lips were not occluded were not linked to the unique contribution of lip movements. Both panels, The shaded areas show the 89% CIs of the respective model. The distributions on the right show the posterior draws of the three models. The black dots represent the mean standardized regression coefficient *b* of the corresponding model. The corresponding bars show the 89% CI. If zero was not part of the 89% CI, the effect was considered significant (*).

We wanted to test whether the unique contribution of lip movements to neural speech tracking (see above, Forward models) yields any behavioral relevance. For this, we also used the behavioral data of the otherwise unanalyzed conditions with a face mask, which were the same number of trials for all participants (see above, Stimuli and experimental design). We fitted Bayesian multilevel models with the averaged unique contribution of lip movements to separately predict the behavioral measures when the speaker wore a face mask or not (Fig. 4). The general formula was as follows:

behavioral measure∼1+unique contribution of lip movements+(1|participant) .

Before doing so, we fitted control models to show the effect of the conditions on the behavioral measures when the lips are occluded. Additional control models to test the effect of the unique contribution of lip movements on the averaged behavioral data without a face mask were also fitted. In all described models, a random intercept was included for each participant to account for repeated measures (single speaker and multispeaker).

Weakly or noninformative default priors of brms were used, whose influence on the results is negligible (Bürkner, 2017, 2018). For model calculation, all numerical variables were *z*-scored, and standardized regression coefficients (*b*) were reported with 89% credible intervals (CIs; i.e., Bayesian uncertainty intervals; McElreath, 2020). In addition, we report posterior probabilities (PP*_b_*_ > 0_) with values closer to 100%, providing evidence that the effect is greater than zero and closer to 0% that the effect was reversed (i.e., smaller than zero). If the 89% CIs for an estimate did not include zero and PP*_b_*_ > 0_ was below 5.5% or above 94.5%, the effects were considered statistically significant.

All models were fitted with a Student’s *t* distribution, as indicated by graphical posterior predictive checks, Pareto $\hat{k}$ diagnostics (Vehtari et al., 2022b), and leave-one-out cross–validation via loo version 2.5.1 (Vehtari et al., 2017, 2022a). Common algorithm-agnostic (Vehtari et al., 2021) and algorithm-specific diagnostics (Betancourt, 2018) showed that all Bayesian multilevel models converged. For all relevant parameters, the convergence diagnostic $\hat{R}<1.01$ and effective sample size >400 indicated that there were no divergent transitions. Figures were created with ggplot2 version 3.4.0 (Wickham, 2016) and ggdist version 3.2.0 (Kay, 2022). Unstandardized *b*'s were used for the fitted values of the models in Figures 3 and 4.

### Data and code availability

Preprocessed data and code are publicly available at GitHub (https://github.com/reispat/av_speech_mask).

## Results

Twenty-nine participants listened to audiobooks with a corresponding video of the speaker and a randomly occurring audio-only distractor. Source-localized MEG responses to acoustic features (spectrogram and acoustic onsets) and lip movements were predicted using forward models (TRFs). We compared the TRFs between the two conditions and evaluated neural tracking of the acoustic features and lip movements. The unique contribution of lip movements was obtained by controlling for acoustic features and was compared between conditions. Using Bayesian multilevel modeling, we predicted the behavioral measures with neural tracking. We also probed the unique contribution of lip movements for their behavioral relevance by predicting the behavioral measures when the lips were occluded with a surgical face mask or not.

### Listening situations with multiple speakers are behaviorally more demanding

Participants performed worse in the multispeaker condition (*M* = 62.93%; SD = 17.34%), compared with the single-speaker condition (*M* = 73.52%; SD = 9.71%; *W* = 73.00; *p* = 0.003; *r*_C_ = 0.64). In the multispeaker condition, subjective difficulty ratings were higher (*M* = 3.67; SD = 0.82) than in the single-speaker condition (*M* = 2.47; SD = 0.71; *W* = 11.50; *p* = 9.00 × 10^−6^; *r*_C_ = −0.95). Engagement was rated higher in the single-speaker condition (*M* = 3.91; SD = 0.74) compared with the multispeaker condition (*M* = 3.72; SD = 0.85; *W* = 29.00; *p* = 0.024; *r*_C_ = 0.62). Overall, behavioral data showed that in the multispeaker condition, participants performed worse, reported the task to be more difficult, and were less motivated (Fig. 1*B*).

### Neural responses to lip movements are enhanced in a multispeaker setting

First, we analyzed the neural responses to acoustic and visual speech features by statistically comparing the corresponding TRFs between the single- and multispeaker conditions within their respective fROIs (Fig. 2*A*). The spectrogram TRFs showed a significant difference between conditions, with three clusters extending from early (30–110 ms; *t* = −5.26; *p* = 0.0001; *d* = −0.81), middle (160–290 ms; *t* = −3.78; *p* = 0.003; *d* = −1.00), and late (310–470 ms; *t* = −5.58; *p* = 0.0001; *d* = −1.02) time ranges. Grand-average TRF peaks are more pronounced in the single-speaker condition, with two peaks at 70 and 180 ms. While the first peak is also present in the multispeaker condition, the second peak appeared 50 ms earlier than the single-speaker setting. The latter peak caused the largest differences in the magnitudes of the TRFs, which are most prominent in the right hemisphere of the fROI.

The TRFs to acoustic onsets showed a significant difference between single- and multispeaker speech, with three clusters extending from early (−20 to 80 ms; *t* = −5.39; *p* < 0.001; *d* = −1.10; Fig. 2*A*), mid (120–140 ms; *t* = −4.54; *p* = 0.004; *d* = −1.43), and mid-late (190–260 ms; *t* = −6.11; *p* < 0.001; *d* = −1.13) time windows. The TRFs showed two peaks at 70 and 190 ms in the single-speaker condition. Similar to the spectrogram TRFs, the first peak in the multispeaker condition is at the same time point as in the single-speaker condition, and the second peak is 50 ms earlier. The magnitude differences across peaks and hemispheres are not substantially different.

TRFs to lip movements show an opposite pattern to the TRFs to acoustic features, with stronger processing in the multispeaker condition. Significant condition differences in the TRFs to lip movements between single- and multispeaker speech were found, with four clusters extending from early (−20 to 70 ms; *t* = 4.41; *p* = 0.0005; *d* = 0.86; Fig. 2*A*), mid (140–270 ms; *t* = 3.97; *p* = 0.001; *d* = 0.88), mid-late (290–330 ms; *t* = 3.34; *p* = 0.01; *d* = 0.91), and late (420–460 ms; *t* = 3.90; *p* = 0.002; *d* = 0.90) time windows. The latencies of the peaks were later in general (160 and 290 ms), as compared with the acoustic TRFs, which is also in line with the longer duration for a stimulus to reach the visual system (Thorpe et al., 1996; VanRullen and Thorpe, 2001). In the single-speaker condition, the peaks are delayed by 10 ms compared with the multispeaker condition, and magnitude differences are most prominent in the first peak and left hemisphere.

Our initial analysis showed that neural responses to acoustic features are stronger when speech is clear. In contrast, neural responses to lip movements were enhanced in a multispeaker environment. The stronger processing of lip movements suggests a greater reliance on the lips of a speaker when speech is harder to understand.

### The cocktail party diametrically affects acoustic and visual neural speech tracking

So far, the TRF results indicate a stronger neural response to lip movements and a weaker one to acoustic features when there is more than one simultaneous speaker. We also wanted to answer the question whether neural tracking of audiovisual speech features differs between the single-speaker and multispeaker conditions in their respective fROIs (Fig. 2*B*). Acoustic neural tracking in the nonaveraged acoustic fROI showed a significant condition difference in the left (*t* = −8.04; *p* < 0.001; *d* = −1.47) and right (*t* = −9.26; *p* < 0.001; *d* = −1.30) hemispheres. Averaged acoustic neural tracking was higher in the single-speaker condition than in the multispeaker condition (*t*_(28)_ = −8.07; *p* = 8.76 × 10^−9^; *d* = −1.30). Neural tracking of lip movements showed a significant condition difference in the left hemisphere (*t* = 3.83; *p* = 0.037; *d* = 0.51; Fig. 2*B*), with a focal superior parietal area involved. When averaging over sources, neural tracking was higher in the multispeaker condition than in the single-speaker condition (*W* = 114.00; *p* = 0.026; *r*_C_ = 0.48).

Overall, the results showed that neural tracking was enhanced for acoustic features when speech is clear and higher for lip movements when there are multiple speakers. This is in line with the observed neural responses.

### Lip movements enhance neural speech tracking more in multispeaker situations

When there are two speakers, we have so far demonstrated that lip movements are processed more strongly and lead to higher neural tracking compared with one speaker. However, their unique contribution to neural tracking is still unknown, due to the intercorrelation of audiovisual speech features (Chandrasekaran et al., 2009; Daube et al., 2019). To address this, we controlled for the acoustic features so as to obtain the unique contribution of lip movements over and above acoustic speech features. First, the acoustic model was evaluated in the audiovisual fROI (Fig. 2*C*). Acoustic neural tracking was higher in the single-speaker condition than in the multispeaker condition (*t*_(28)_ = −7.20; *p* = 7.68 × 10^−8^; *d* = 1.18). The acoustic model served as a baseline and was subtracted from a combined acoustic + lip model and expressed as percentage change. The obtained unique contribution of lip movements was higher in the multispeaker condition than in the single-speaker condition (*W* = 24.00; *p* = 0.00003; *r*_C_ = 0.89). The unique contribution of lip movements showed high interindividual variability and seemed to follow a bimodal distribution (Fig. 2*C*), which was confirmed by a bimodality coefficient of 0.68 (values >0.555 indicate bimodality; Pfister et al., 2013).

These results strongly indicate that lip movements enhance neural tracking, especially in multitalker speech. However, substantial interindividual variability was observed, with participants showing a unique contribution of lip movements of up to 45.37% in the multispeaker condition, while others showed only a small contribution or no contribution at all. In the next steps, we will probe the behavioral relevance of the unique contribution of lip movements to neural speech tracking by depriving individuals of this source of information.

### Only acoustic neural speech tracking predicts behavior

Having established that listening situations with two speakers affect neural tracking of acoustic and visual speech features in a diametrical way, we were further interested if neural tracking is able to predict the behavioral measures. We calculated Bayesian multilevel models to predict the three behavioral measures (comprehension, difficulty, and engagement; Fig. 1*B*) with the averaged neural tracking of the acoustic and lip models (Fig. 3). In the acoustic model, higher neural tracking was linked to higher comprehension (*b* = 0.29; 89% CI = [0.07, 0.51]; PP*_b_*_ > 0_ = 98.37%; Fig. 3*A*). Lower neural tracking predicted higher difficulty ratings (*b* = −0.50; 89% CI = [−0.72, −0.29]; PP*_b_*_ > 0_ = 0.01%). When neural tracking was high, the engagement ratings were also higher (*b* = 0.12; 89% CI = [0.004, 0.24]; PP*_b_*_ > 0_ = 95.05%).

Neural tracking of lip movements was not related to comprehension (*b* = 0.06; 89% CI = [−0.18, 0.28]; PP*_b_*_ > 0_ = 65.61%; Fig. 3*B*). We also observed no evidence for an effect of the difficulty (*b* = −0.05; 89% CI = [−0.28, 0.18]; PP*_b_*_ > 0_ = 35.63%) or engagement (*b* = 0.09; 89% CI = [−0.08, 0.26]; PP*_b_*_ > 0_ = 80.40%) ratings.

These results indicate that acoustic neural speech tracking predicts behavior: The higher the neural speech tracking, the higher the comprehension and engagement ratings. Lower acoustic neural speech tracking was linked to higher difficulty ratings. In contrast, neural speech tracking of lip movements did not predict behavior.

### Stronger unique contribution of lip movements predicts behavioral deterioration when lips are occluded

Given the finding that lip movements enhance neural speech tracking (Fig. 2*C*), we were interested in whether this unique contribution to neural speech tracking is behaviorally relevant. To do so, we also used the behavioral data from the otherwise unanalyzed conditions in which the mouth was occluded by a surgical face mask (see the center of Fig. 4*A* for example stimuli). Given that critical visual information is missing in these conditions, individuals who show a strong unique contribution of lip movements on a neural level should show poorer behavioral outcomes. An initial analysis showed that the effect of the conditions with a surgical face mask on behavior followed a similar pattern as those with nonoccluded lips (Fig. 1*B*): Comprehension was worse in the multispeaker condition (*b* = −0.77; 89% CI = [−1.13, −0.41]; PP*_b_*_ > 0_ = 0.07%). Subjective difficulty ratings were also higher in the multispeaker condition (*b* = −0.77; 89% CI = [−1.13, −0.41]; PP*_b_*_ > 0_ = 0.07%). However, there was no effect of the conditions with a surgical face mask on the engagement ratings (*b* = −0.77; 89% CI = [−1.13, −0.41]; PP*_b_*_ > 0_ = 0.07%).

While the effects on a solely behavioral level seem not to differ substantially when the lips are occluded or not, predicting the behavioral measures with the unique contribution of lip movements showed the expected outcome (Fig. 4*A*): Participants that had a higher unique contribution of lip movements in terms of neural tracking showed a decline in comprehension (*b* = −0.27; 89% CI = [−0.49, −0.06]; PP*_b_*_ > 0_ = 2.21%) and reported the task to be more difficult (*b* = 0.25; 89% CI = [0.01, 0.51]; PP*_b_*_ > 0_ = 95.41%). The engagement ratings did not yield an effect (*b* = 0.05; 89% CI = [−0.07, 0.18]; PP*_b_*_ > 0_ = 76.14%).

Interestingly, we were not able to establish a link between the unique contribution of lip movements to the behavioral data when the lips were not occluded (Fig. 4*B*). Comprehension (*b* = −0.05; 89% CI = [−0.28, 0.17]; PP*_b_*_ > 0_ = 36.09%), difficulty (*b* = 0.04; 89% CI = [−0.19, 0.28]; PP*_b_*_ > 0_ = 60.86%), and engagement (*b* = 0.06; 89% CI = [−0.08, 0.19]; PP*_b_*_ > 0_ = 76.64%) were not linked to the unique contribution of lip movements.

Taken together, these findings support a behavioral relevance of the unique contribution of lip movements. Individuals that have a higher unique contribution of lip movements on a neural level performed worse and reported the task to be more difficult when the mouth of the speaker was covered by a surgical face mask.

## Discussion

The method of neural speech tracking is widely used to study the neural processing of continuous speech, though primarily with audio-only stimuli (Di Liberto et al., 2015; Keitel et al., 2018; Brodbeck et al., 2018a; Chalas et al., 2022). Recent studies have used audiovisual speech paradigms, but without directly modeling visual speech features and their temporal dynamics (Golumbic et al., 2013; Crosse et al., 2016b). In this study, we first show the temporal dynamics and cortical origins of TRFs obtained from lip movements in an audiovisual setting with one or two speakers. Similar to Brodbeck et al. (2018a), neural responses to acoustic features in the two-speaker paradigm were generally weaker. In both acoustic features, we observed that the second peak was 50 ms earlier when there were two speakers. Similar temporal differences in TRFs were also observed in normal-hearing individuals in a selective attention speech paradigm (Kaufman and Zion Golumbic, 2023), as well as in cochlear implant users, where the attended speech was showing enhanced earlier responses compared with ignored speech (Kraus et al., 2021). The TRFs to lip movements showed an opposite pattern, with an enhanced magnitude in the multispeaker condition (Fig. 2*A*) and with substantially later peaks compared with the TRF to acoustic features. This is in line with Bourguignon et al. (2020), where initial TRF peaks at 115 and 159 ms were shown from two significant sources, overlapping with our involved parietal and occipital sources (Fig. 1*C*). However, the TRFs in their work were modeled to lip movements from silent videos, which precludes a comparison between different listening situations. The finding that the peaks of TRFs occur later for lip movements than for auditory features seems counterintuitive, as the visual stimulus usually precedes the auditory stimulus (van Wassenhove et al., 2005). One possible reason for this could be that visual stimuli require a longer period of time to reach the visual system compared with auditory stimuli (Thorpe et al., 1996; VanRullen and Thorpe, 2001), thus leading to a later neural response when modeled using TRFs. For TRFs to lip movements, we also observed a stronger contribution of left parietal and occipital regions, especially when contrasting the first peak. A possible explanation for this lateralization could be due to asymmetries in the processing of lip movements, where previous studies showed a left-hemispheric advantage (Campbell et al., 1996; Nicholls and Searle, 2006).

Our findings also strengthen the argument that TRFs to visual speech are quantitatively different from TRFs to acoustic speech (for an analysis based on coherence, see Park et al., 2016). In this study, however, we were not able to completely rule out the contribution of auditory speech to modeled TRFs to lip movements, since an audiovisual paradigm was used. In future studies, a visual-only condition should also be incorporated to further compare the differences between TRFs derived from lip movements in an audiovisual or visual-only condition.

Based on the source-localized neural tracking, we determined fROIs via a data-driven approach—separately for the acoustic features and lip movements (Fig. 1*C*). The fROIs for the acoustic speech features involved sources along temporal, parietal, and posterior frontal regions, covering regions that are related to speech perception (Franken et al., 2022). Previous studies source-localized TRFs in audio-only settings, though commonly restricting the analysis to temporal regions (Brodbeck et al., 2018a; Kulasingham et al., 2020). The fROIs for the lip movements involved parietal and occipital regions, in line with previous studies that source-localized the neural tracking of lip movements (Hauswald et al., 2018; Bourguignon et al., 2020; Aller et al., 2022). We also observed neural tracking of lip movements in temporal regions (similar to Park et al., 2016) but with less involvement of the primary visual cortex and prominent only in the single-speaker condition. Due to our approach of defining our fROIs based on the multispeaker condition, we minimized the involvement of auditory regions in the lip fROIs.

When analyzing neural speech tracking in the acoustic fROIs, we showed a large effect with enhanced tracking in the single-speaker condition compared with the multispeaker condition (Fig. 2*B*). Using phase consistency as a neural tracking method, and not statistically tested, a large difference in neural tracking between single- and multispeaker speech was shown by Golumbic et al. (2013). We were not able to identify further studies that presented such a statistical contrast, which could be due to the general focus on neural tracking of attended versus unattended speech, especially to decode auditory attention (Mirkovic et al., 2015; O’Sullivan et al., 2015; Schäfer et al., 2018; Ciccarelli et al., 2019; Geirnaert et al., 2021). On a group level, the neural tracking of lip movements showed an enhancement in the multispeaker condition (Fig. 2*B*). When comparing the involved sources of the corresponding lip fROI, we found a medium effect in the left superior parietal cortex. This is in line with Park et al. (2016), showing an effect in the left occipital and parietal cortex when comparing two similar conditions to our design (“AV congruent vs All congruent”), although after partializing out auditory-related coherence. A possible explanation for the strong focality of our left superior parietal effect could be due to the used TFCE method with a small default step size of 0.1, leading to a downweighting of spatially larger clusters (Smith and Nichols, 2009). When we averaged the neural tracking of lip movements, we observed interesting patterns, with participants showing no meaningful neural tracking (i.e., close to zero or negative correlations) when there was one speaker, but when speech became challenging, their neural tracking reached positive values. Notably, this pattern was reversed for some participants, suggesting that not all of them used the lip movements in the same manner. To investigate this further, eye tracking should be used to identify which face regions participants fixated when attending audiovisual speech (Rennig and Beauchamp, 2018) or to additionally incorporate a recently proposed phenomenon termed “ocular speech tracking” (Gehmacher et al., 2024). In this study, we cannot rule out that during challenging speech, participants fixated the mouth area stronger, thus contributing to enhanced neural tracking. However, previous eye tracking research has shown that individuals gaze at talking (Gurler et al., 2015) and also nontalking faces (Peterson and Eckstein, 2013; Mehoudar et al., 2014) in a highly individual manner, which is putatively incorporated in our findings of high interindividual variability.

We first compared the neural tracking of audiovisual speech between single-speaker and multispeaker conditions in an isolated manner. Due to the aforementioned intercorrelation of audiovisual speech features (Chandrasekaran et al., 2009; Daube et al., 2019), this approach could not rule out any acoustic contributions to the neural tracking of lip movements or vice versa. To reveal the unique contribution of lip movements and to incorporate regions that are part of models of audiovisual speech perception (Bernstein and Liebenthal, 2014) and multisensory integration (Peelle and Sommers, 2015), we combined both fROIs and controlled for acoustic speech features. Within the TRF framework, we show that lip movements enhance acoustic-controlled neural speech tracking (Fig. 2*C*). A general enhancement was observed for both single- and multispeaker speech, which is in line with behavioral findings that visual speech features enhance intelligibility under clear speech conditions as well (Stacey et al., 2016; Blackburn et al., 2019). When comparing the two conditions, we observed a large effect, showing a higher unique contribution of lip movements in the multispeaker condition. Analogous to behavioral findings in Aller et al. (2022), the unique contribution of lip movements showed high interindividual variability (Fig. 2*C*) and also followed a bimodal distribution: Some individuals showed a strong unique contribution of lip movements, while others showed only a small unique contribution or none at all. Interestingly, one individual even showed a negative influence when adding lip movements to the acoustic model when there was only one speaker. As soon as speech became challenging, that individual showed a contribution of lip information. Previous research on audiovisual speech processing showed that interindividual differences are related to visual attention (Tiippana et al., 2004), availability of attentional resources (Alsius et al., 2005), or individual preference for auditory or visual stimuli (Schwartz, 2010), which are potential factors that could explain our observed differences. Overall, our findings are in line with the beneficial effects of visual speech when listening is challenging (Sumby and Pollack, 1954; Grant and Seitz, 2000; Ross et al., 2007; Remez, 2012).

Using Bayesian multilevel modeling, we established a link between neural speech tracking and behavior. We show that higher acoustic neural tracking is related to higher comprehension (Fig. 3*A*), a finding also reported in a study that used vocoded speech (Chen et al., 2023). We also show that higher acoustic neural tracking is related to lower difficulty ratings. This is in line with a study that showed a positive relationship between speech intelligibility ratings and acoustic neural tracking, though using speech-in-noise (Ding and Simon, 2013). Higher engagement ratings were associated with higher acoustic neural tracking—in contrast to Schubert et al. (2023)—showing no relationship between the two measures. Our findings suggest that enhanced neural speech tracking of acoustic features is related to a lower listening effort, where cognitive demand and motivation are the key contributors (Peelle, 2018). Interpreting the relationship with comprehension performance and lower difficulty ratings as cognitive demand and that with motivation as engagement, our results putatively reflect a neural proxy of listening effort.

We were not able to establish any link between the neural tracking of lip movements and the behavioral measures. It is important to note here that the analyzed neural tracking of lip movements was not yet controlled for speech acoustics (Gillis et al., 2022), which could confound any relationship with behavior. A recent MEG study impressively showed that the neural tracking of acoustic speech features can explain cortical responses to higher-order linguistic features, such as phoneme onsets (Daube et al., 2019). It is important to note that this caveat also applies to the observed relationship between acoustic neural tracking and behavior, and it cannot be ruled out that this relationship is driven by these higher-order features. Further audiovisual speech studies, in which linguistic features are also modeled, are necessary.

The COVID-19 pandemic established the use of face masks on a global scale (Feng et al., 2020). However, it has been demonstrated that covering the mouth has adverse effects on behavioral measures, such as speech perception (Rahne et al., 2021). On a neural level, Haider et al. (2022) showed that surgical face masks impair the neural tracking of acoustic and higher-order segmentational speech features. In a follow-up study, Haider et al. (2024) incorporated lip movements in the analysis and showed that face masks primarily impact speech processing by blocking visual speech rather than by acoustic degradation. Here, we establish a relationship between behavioral measures and the unique contribution of visual speech on neural tracking, which has not yet been shown. When the speaker wore a surgical face mask, individuals that show a higher unique contribution of lip movements displayed lower comprehension and higher difficulty ratings. Strikingly, no effect was found when the speaker did not wear a surgical face mask. Further studies with larger sample sizes are needed to disentangle the potential influence of experimental conditions on this relationship, e.g., using Bayesian mediation analysis (Yuan and MacKinnon, 2009; Nuijten et al., 2015). Overall, our results suggest that individuals who use lip movements more effectively show behavioral deterioration when visual speech is absent.

The current study provides evidence for the substantial interindividual variability in the unique contribution of lip movements to neural speech tracking and its relationship to behavior. First, we show that neural responses to lip movements are more pronounced when speech is challenging, compared with when speech is clear. We show that lip movements effectively enhance neural speech tracking in brain regions related to audiovisual speech, with high interindividual variability. Furthermore, we demonstrate that the unique contribution of lip movements is behaviorally relevant. Individuals that show a higher unique contribution of lip movements show lower comprehension and rate the task to be more difficult when the speaker wears a surgical face mask. Remarkably, this relationship is completely absent when the speaker did not wear a mask. Our results provide insights into the individual differences in the neural tracking of lip movements and offer potential implications for future clinical and audiological settings to objectively assess audiovisual speech perception, such as in populations where traditional task-based assessments cannot be meaningfully conducted.

## Synthesis

Reviewing Editor: Christine Portfors, Washington State University Decisions are customarily a result of the Reviewing Editor and the peer reviewers coming together and discussing their recommendations until a consensus is reached. When revisions are invited, a fact-based synthesis statement explaining their decision and outlining what is needed to prepare a revision will be listed below. The following reviewer(s) agreed to reveal their identity: Aaron Nidiffer. Note: If this manuscript was transferred from JNeurosci and a decision was made to accept the manuscript without peer review, a brief statement to this effect will instead be what is listed below.

Thank you to the authors for addressing the previous comments completely by either making meaningful changes to the manuscript or providing thoughtful reasoning to their decisions for each point brought up.
